# *VPS35* pathogenic mutations confer no dominant toxicity but partial loss of function in *Drosophila* and genetically interact with *parkin*

**DOI:** 10.1093/hmg/ddv322

**Published:** 2015-08-06

**Authors:** Bilal R. Malik, Vinay K. Godena, Alexander J. Whitworth

**Affiliations:** Department of Biomedical Sciences, University of Sheffield, Sheffield S10 2TN, UK

## Abstract

Mutations in *VPS35* (*PARK17*) cause autosomal dominant, late onset Parkinson's disease (PD). VPS35 forms a core component of the retromer complex that mediates the retrieval of membrane proteins from endosomes back to either the Golgi or plasma membrane. While aberrant endosomal protein sorting has been linked to several neurodegenerative diseases, the mechanisms by which *VPS35* mutations and retromer function contribute to PD pathogenesis are not clear. To address this, we generated transgenic *Drosophila* that express variant forms of human *VPS35* found in PD cases and the corresponding variants of the *Drosophila* ortholog. We did not find evidence of dominant toxicity from any variant form including the pathogenic D620N mutation, even with aging. However, assessing the ability of Vps35 variants to rescue multiple *vps35-*mutant phenotypes, we found that the D620N mutation confers a partial loss of function. Recently, VPS35 has been linked to the formation of mitochondria-derived vesicles, which mediate the degradation of mitochondrial proteins and contribute to mitochondrial quality control. This process is also promoted by two other PD-lined genes *parkin* (*PARK2*) and *PINK1* (*PARK6*). We demonstrate here that *vps35* genetically interacts with *parkin* but interestingly not with *pink1*. Strikingly, Vps35 overexpression is able to rescue several *parkin*-mutant phenotypes. Together these findings provide *in vivo* evidence that the D620N mutation likely confers pathogenicity through a partial loss of function mechanism and that this may be linked to other known pathogenic mechanisms such as mitochondrial dysfunction.

## Introduction

Parkinson's disease (PD) is a common neurodegenerative disorder that is principally characterized by the progressive loss of motor control and degeneration of dopaminergic neurons in the basal ganglia. Although current treatments provide temporary symptomatic relief, their efficacy declines and the condition is currently incurable. A greater understanding of the pathogenic mechanisms would allow the development of more rational disease-modifying interventions. In recent years, genetic analysis of Mendelian forms of PD has shed tremendous light on the molecular players in pathogenesis. Recently, a missense mutation, Asp620Asn (D620N), in *VPS35* (*PARK17*) was identified in a number of families with dominantly inherited, late-onset PD (1,2). Additional variants, including P316S, R524W and L774M, have also been found in sporadic patients, but their presence also in unaffected controls has rendered the pathogenicity of these uncertain (1,2).

VPS35 is a central component of the highly conserved retromer complex that mediates the retrieval of transmembrane cargo proteins from endosomes (3,4). Cargo-selective sorting is critical for regulating the proper sub-cellular destination of endosomal proteins. The most widely characterized role for retromer is in the recycling of transport proteins, such as the cation-independent mannose 6-phosphate receptor (5) or Wnt transport protein Wntless (6–8), back to the trans-Golgi network (TGN). More recently, the cargo-selective retromer complex has also been shown to recruit the actin nucleation promoting Wiskott–Aldrich syndrome and SCAR homolog (WASH) complex to endosomes (9–11), mediated directly via VPS35 (12). This promotes the recycling of specific cargo, such as the β2-adrenergic receptor and the glucose transporter GLUT1, directly back to the plasma membrane (13).

Current literature on the impact of the pathogenic D620N mutation on retromer function presents conflicting results on whether endosome-to-TGN and/or endosome-to-plasma membrane recycling is affected (14–16), or in fact whether VPS35 variants confer dominant toxicity or are functionally hypomorphic (14,17,18). However, recent studies have revealed that the pathogenic D620N mutation inhibits VPS35 binding to the WASH complex, consistent with a partial loss of function (15,16). Nevertheless, much still remains to be clarified on the pathogenic nature of *VPS35* mutations and the mechanism(s) by which they cause neurotoxicity, especially *in vivo*.

A surprising development in the emerging complexity of retromer function was the report that Vps35 appears to mediate the formation and trafficking of mitochondria-derived vesicles (MDVs) (19). Similar to endosomes, these MDVs are also cargo selective and have a variety of destinations, including peroxisomes and lysosomes (19,20). The precise role of these MDVs is currently uncertain but is likely to provide a route for mitochondrial component degradation and thus a mechanism of mitochondrial quality control (20,21). Moreover, recent evidence indicates that two genes causative for autosomal-recessive parkinsonism, *PINK1* (*PARK6*) and *parkin* (*PARK2*), promote the formation of cargo-specific MDVs targeted for lysosomal degradation (22), implicating this process in the pathogenesis of PD.

Here, we sought to develop a simple *in vivo* model of *VPS35*-related PD using *Drosophila* in order to address the relative pathogenicity of known variants and to shed light on the possible pathogenic mechanisms. Expressing variant forms of human *VPS35* or the equivalent mutations in *Drosophila vps35*, we found no evidence of dominant toxicity, even with ageing. In contrast, all variants functioned similar to wild-type (WT), being able to substantially rescue *vps35*-mutant phenotypes including one assay of endosome-to-TGN recycling. However, some assays showed that mutant Vps35 functioned less well than WT, suggesting that they may be hypomorphic. Interestingly, we observed a strong genetic interaction between *vps35* and *parkin*, where double heterozygotes presented a synergistic loss of dopaminergic neurons, an enhanced climbing defect that was exacerbated with age and a striking sensitivity to paraquat. In genetic rescue experiments, *vps35* overexpression substantially suppressed several *parkin*-mutant phenotypes but not vice versa. Thus, our genetic studies suggest that *vps35* variants associated with PD are likely to confer pathogenicity by haploinsufficiency rather than a toxic gain of function and suggest that mitochondrial quality control may be an important pathway in *vps35*-meditated pathogenesis.

## Results

### No dominant toxicity from *vps35* pathogenic variants

**Figure 1.**
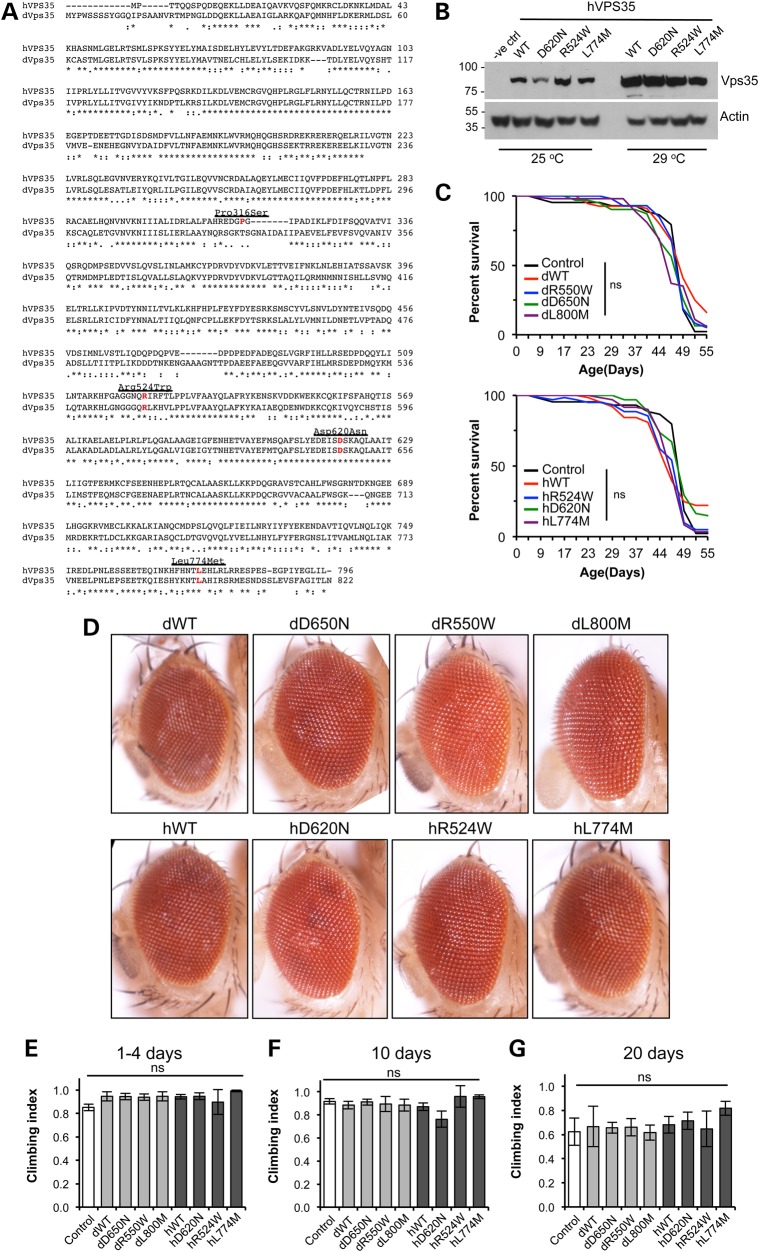
No dominant toxicity of human or *Drosophila* Vps35 variants. (**A**) ClustalW2 Alignment of Vps35 amino acid sequence from human (hVps35) and *Drosophila* (dVps35) with the missense mutations highlighted. (**B**) Immunoblot of human Vps35 transgenes raised at different temperatures to monitor relative expression levels. (**C**) Transgene expression does not affect lifespan when expressed ubiquitously with *da*-*GAL4* [all not significant (ns) compared with control, Log-rank test, *n* = 40–100 animals]. (**D**) No overt phenotype is seen when the transgenes were expressed in the compound eye by *GMR*-*GAL4*. (**E**–**G**) Ubiquitous expression does not affect climbing ability with age [all not significant (ns) compared with control]. *n* = 25–40 (E), 20–40 (F) and 10–40 (G). Histograms show mean ± SEM. Statistical analysis was one-way analysis of variance (ANOVA) with Bonferroni's correction. Control genotypes are heterozygous *GAL4* driver line alone.

Vps35 is highly conserved with human and *Drosophila* protein homologs sharing 61% identity and 78% similarity (Fig. 1A). To investigate the pathogenicity of *vps35* mutations *in vivo*, we generated transgenic *Drosophila* that express variant forms of human *VPS35* or, where these residues are conserved, equivalent variants of *Drosophila vps35*. Since residues R524, D620 and L774 are conserved in *Drosophila* Vps35, while P316 is not, we focussed our analysis on these mutations. Transgenic *Drosophila* were generated that express WT and variant forms of either human or *Drosophila vps35* under the control of the GAL4/UAS system. Here, we designate the variants of human VPS35 as hWT, hD620N, h524W and hL774M, while the *Drosophila* equivalent variants are designated dWT, D650N, dR550W and dL800M.

Expression of the human Vps35 variants was verified by immunoblotting (Fig. 1B). Reliable antibodies against *Drosophila* Vps35 are not currently available, but we assume approximately equivalent expression levels due to the site-specific integration of the transgenes.

Since *VPS35* mutants cause autosomal dominant PD, we first assessed whether expression of these transgenes caused dominant phenotypes associated with neuronal toxicity. We analysed effects on lifespan, retinal degeneration and locomotor activity; however, no apparent toxicity was observed from expression of all the transgenes (Fig. 1C–E). Moreover, no defects in motor ability appeared even after ageing (Fig. 1F and G). To enhance expression to physiologically relevant levels (see below), we also raised and aged animals at 29°C. Under these conditions, transgenic animals showed a rather modest, albeit significant, impairment to lifespan compared with controls, but all Vps35 variants showed equivalent effects (Supplementary Material). However, there was still no significant effect on climbing behaviour compared with controls at either 1–4 days or 10 days old, across all transgenic lines.

### Vps35 variants confer partial loss of function

Since no phenotypes were observed from overexpression in a WT background, it was essential to determine whether the transgenes faithfully replicated Vps35 functionality. A definitive test is to determine whether transgene expression can substitute for endogenous *vps35* function by rescuing mutant phenotypes. Loss of function mutations in *Drosophila vps35* cause severe developmental defects resulting in larval-stage lethality and the appearance of melanotic masses in various homozygous and transheterozygous combinations (6–8). All transgenes were expressed in a *vps35* transheterozygous-mutant background (*vps35^MH20^/vps35^E42^*), and viability was determined. Surprisingly, despite using a strong ubiquitous driver, animals raised under standard conditions (25°C) showed no rescue of viability with any of the Vps35 variants (data not shown), so we raised animals at a higher temperature (29°C) to increase transgene expression levels (Fig. 1B). Expression of all variants of *Drosophila vps35* was now sufficient to prevent the appearance of melanotic masses and fully rescue the developmental lethality, with progeny emerging in expected proportions. However, while the human *VPS35* variants rescued mutant viability to late pupal stage, adults failed to eclose indicating that human VPS35 protein cannot fully substitute for the *Drosophila* orthologue. In light of these results, all subsequent rescue experiments were performed with animals grown at 29°C.

**Figure 2.**
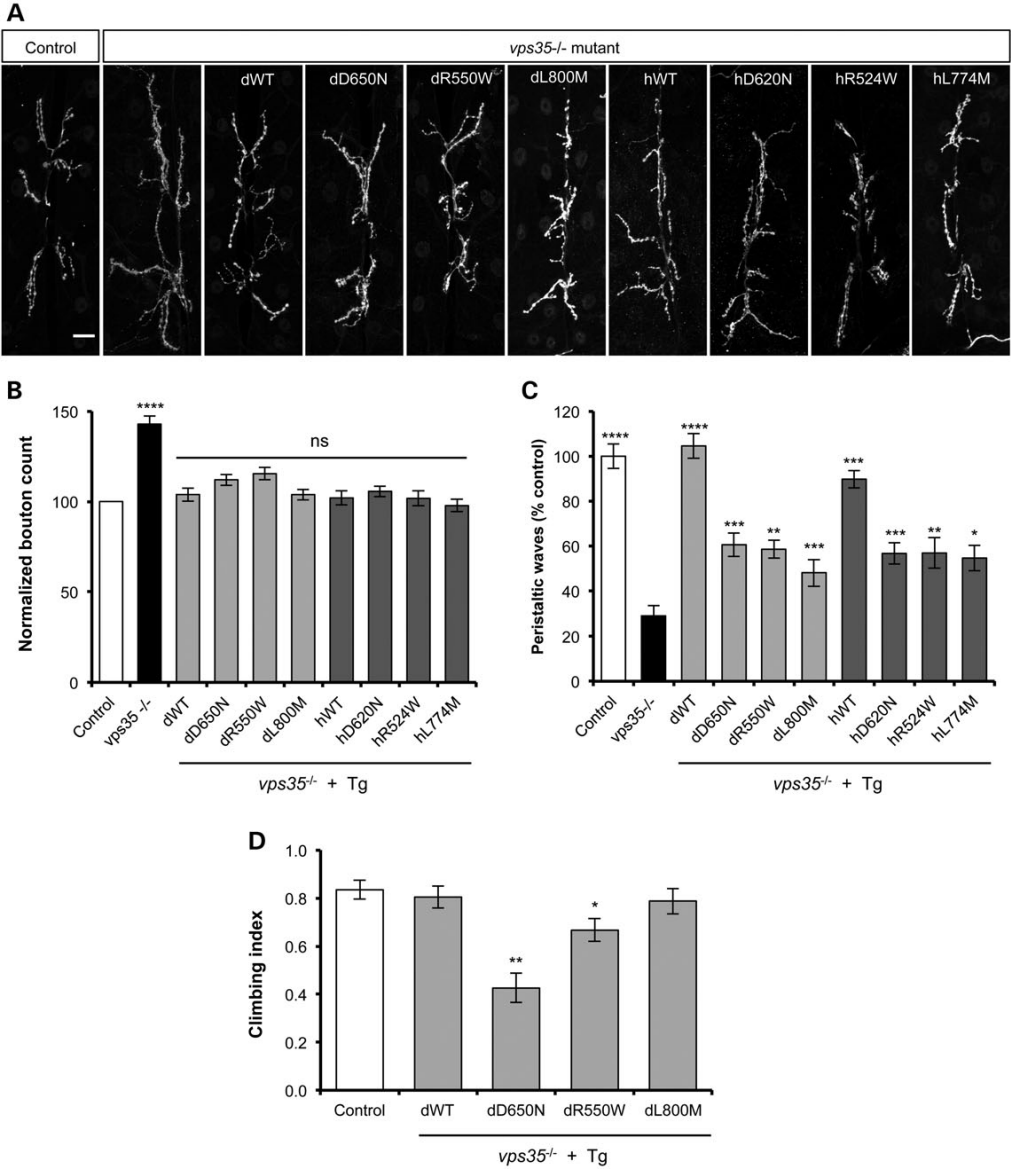
Variable effects of Vps35 variants to rescue synaptic overgrowth and locomotor phenotypes. (**A**) Anti-HRP immunofluorescence reveals ‘overgrown’ NMJs in *vps35* mutants. Scale bar = 10 µm. (**B**) Quantification shows significantly more synaptic boutons in *vps35* mutants. *n* = 17–34 animals. All transgenes (Tg) provide a significant complete rescue of this phenotype. (**C**) *vps35* mutants display defective larval locomotion, measured by the number of peristaltic waves per 2 min. *n* = 5–14 animals. All transgenes provide some rescue of this phenotype, but the variant forms are substantially less effective here. (**D**) Climbing assay of *vps35* mutants expressing *Drosophila vps35* transgenes. *n* = 30–46 animals. All animals were raised at 29°C. Statistical analysis: ns = not significant, *P < 0.05, **P < 0.01, ***P < 0.001 and ****P < 0.0001; one-way ANOVA with Bonferroni's correction. Control genotype is heterozygous *da-GAL4* driver line alone.

*vps35* mutants show defective development of the neuromuscular junction (NMJ), caused in part by aberrant bone morphogenic protein signalling. Loss of *vps35* leads to the formation of supernumerary boutons (Fig. 2A and B). This developmental defect in the NMJ is accompanied by a dramatic reduction in the larval locomotion (Fig. 2C). Expression of all variants of *Drosophila* and human Vps35 was able to completely restore normal NMJ formation (Fig. 2A and B). However, the larval locomotion defect showed a more variable response (Fig. 2C). While the expression of WT dVps35 could completely restore locomotion to control levels, expression of WT hVps35 could also substantially rescue this deficit. In contrast, the missense variants of both dVps35 and hVps35 could only partially substitute for loss of *vps35* for this phenotype.

The ability of the *Drosophila* transgenes to successfully rescue *vps35*-mutant viability allowed us to further test the relative functionality of the mutant variants using the climbing assay. This higher-order behavioural test revealed that expression of the dWT or dL800M variant enabled comparable climbing capacity with controls (Fig. 2D). In contrast, the dD650N pathogenic mutation, and to a lesser extent the dR550W variant, produced a clear climbing defect (Fig. 2D).

**Figure 3.**
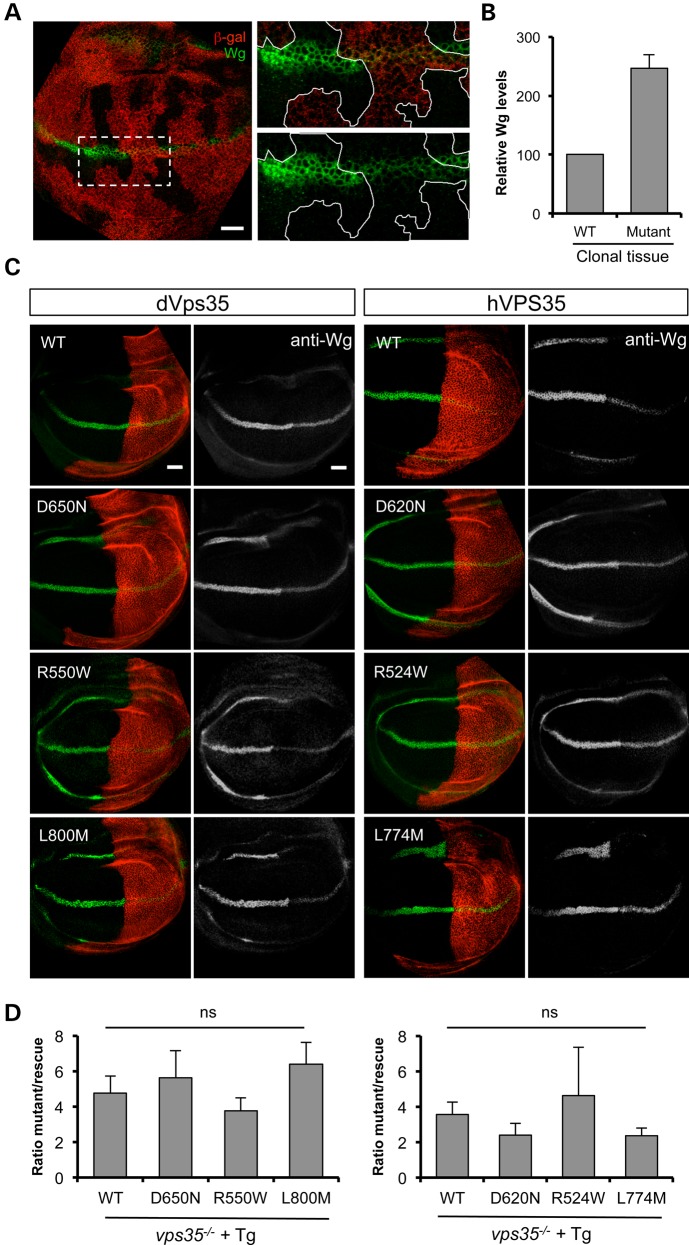
All transgenes rescue Wg secretion in *vps35* mutants. (**A**) Anti-Wg immunostaining (green) reveals intracellular accumulation of Wg in FLP/FRT clones of *vps35^MH20^* in wing imaginal discs. Loss of anti-β-gal immunostaining (red) marks the clones lacking *vps35*. (**B**) Quantification of relative anti-Wg immunostaining in *vps35* clones compared with neighbouring WT tissue. Histogram shows mean ± SEM. *n* = 15 ROIs from nine animals. (**C**) Wing imaginal discs from *vps35* null mutants in which *hh*-*GAL4* drives expression of Vps35 transgenes in the posterior half along with an red fluorescent protein marker (red). (**D**) Quantification of the relative immunofluorescence from anterior (mutant) versus posterior (rescued) segments of Wg expression domain. Histogram shows mean ± SEM. *n* = 5–6 discs per genotype, analysed by one-way ANOVA with Bonferroni's correction (ns = not significant). Scale bar = 10 µm.

The retromer function of Vps35 has been shown to regulate the secretion of wingless (Wg) in the larval wing imaginal disc. Failure of the retromer complex to recycle the Wg transporter protein Wntless leads to an intracellular accumulation of Wg and concomitant signalling defects. This can be observed by increased immunostaining of Wg in *vps35*-mutant clones compared with neighbouring WT tissue (Fig. 3A and B), as previously reported. To further test the relative function of Vps35 variants, we sought to determine how well they could restore Wg levels in *vps35-*mutant wing discs. To allow comparison within the same tissue, we analysed wing discs from *vps35*-mutant animals in which Vps35 transgene expression was restricted to the posterior compartment via induction with *hedgehog-GAL4* (Fig. 3C). While the expression of all transgenes reduced the aberrant Wg levels, quantification across multiple samples showed that all variants restored the levels of Wg to a similar degree (Fig. 3D).

Taken together, the preceding results indicate that not only do the Vps35 variants appear to not confer any major dominant toxicity, but all variants appear to function largely normally in endosome-to-plasma membrane recycling. However, while NMJ structural defects are restored by all Vps35 variants, in functional locomotor assays, the pathogenic variants consistently reveal a partial loss of function.

### *Vps35* genetically interacts with *parkin* but not *pink1*

Since our analysis of transgenic Vps35 variants provided no evidence for a toxic pathologic mechanism in our model system, we next sought to determine whether *vps35* mutations may influence other pathogenic causes. One putative link came from the observations that Vps35 appears to play a role in the generation of MDVs. Importantly, this process was also recently linked to *PINK1* and *parkin*, two autosomal-recessive PD genes strongly implicated in mitochondrial quality control. MDVs have been proposed to function as part of the quality control mechanism as some vesicles carrying oxidised protein cargoes are trafficked to the lysosome for degradation. Thus, we tested whether *vps35* may genetically interact with *parkin* and *pink1*.

**Figure 4.**
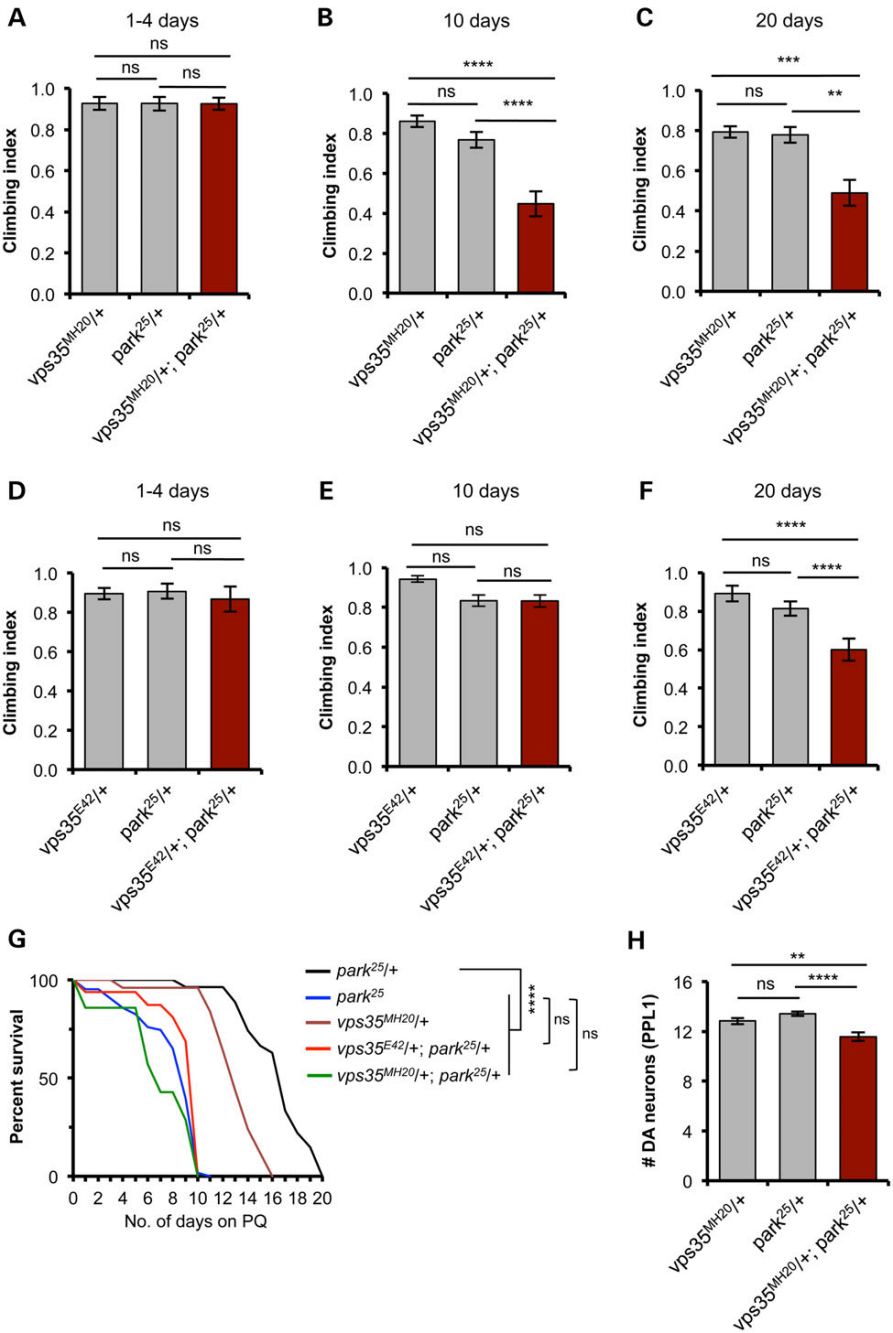
*vps35* genetically interacts with *parkin*. (**A–F**) Climbing ability was assessed at different ages in animals single and double heterozygous for null alleles of *parkin* (*park^25^*) and two independent null alleles of *vps35*, *vps35^MH20^* (A–C) and *vps35^E42^* (D–F). *n* = 38–48 (A), 42–75 (B), 36–54 (C), 50–63 (D), 63–80 (E) and 44–64 (F). (**G**) Survival analysis of single and double heterozygotes, alongside *park^25^* homozygotes, exposed to 5 mm paraquat (PQ). *n* = 8–60 animals. (**H**) Analysis of dopaminergic neuron loss in the PPL1 cluster in 30-day-old animals. *n* = 17, 22 and 16, respectively. Histograms show mean ± SEM. Statistical analysis: ns = not significant, **P < 0.01, ***P < 0.001 and ****P < 0.0001; one-way ANOVA with Bonferroni's correction, or Log-rank test for survival.

Animals heterozygous for *vps35* or *parkin* show no phenotype compared with WT; however, we found a significantly worse climbing ability in *vps35/+;parkin/+* double heterozygotes, analysing two independent alleles of *vps35* (Fig. 4A–F). Interestingly, this synergy was age dependent; not apparent in young animals, but worsening in older animals. These observations suggest that *parkin* and *vps35* may partly act in a common pathway, and one that is an age-related process.

Oxidative stress is a prominent feature of PD pathology and of normal ageing. Many models of PD show sensitivity to oxidative stressors such as paraquat, including *Drosophila parkin* mutants (Fig. 4G). Similar to the locomotor assay, while the single heterozygotes were relatively insensitive, we found a synergistic sensitivity of *vps35/+;parkin/+* double heterozygotes to paraquat exposure, comparable in severity with *parkin* homozygotes (Fig. 4G). Interestingly, we also saw a genetic interaction of *vps35* and *parkin* in the degeneration of DA neurons of the PPL1 cluster after 30 days (Fig. 4H).

**Figure 5.**
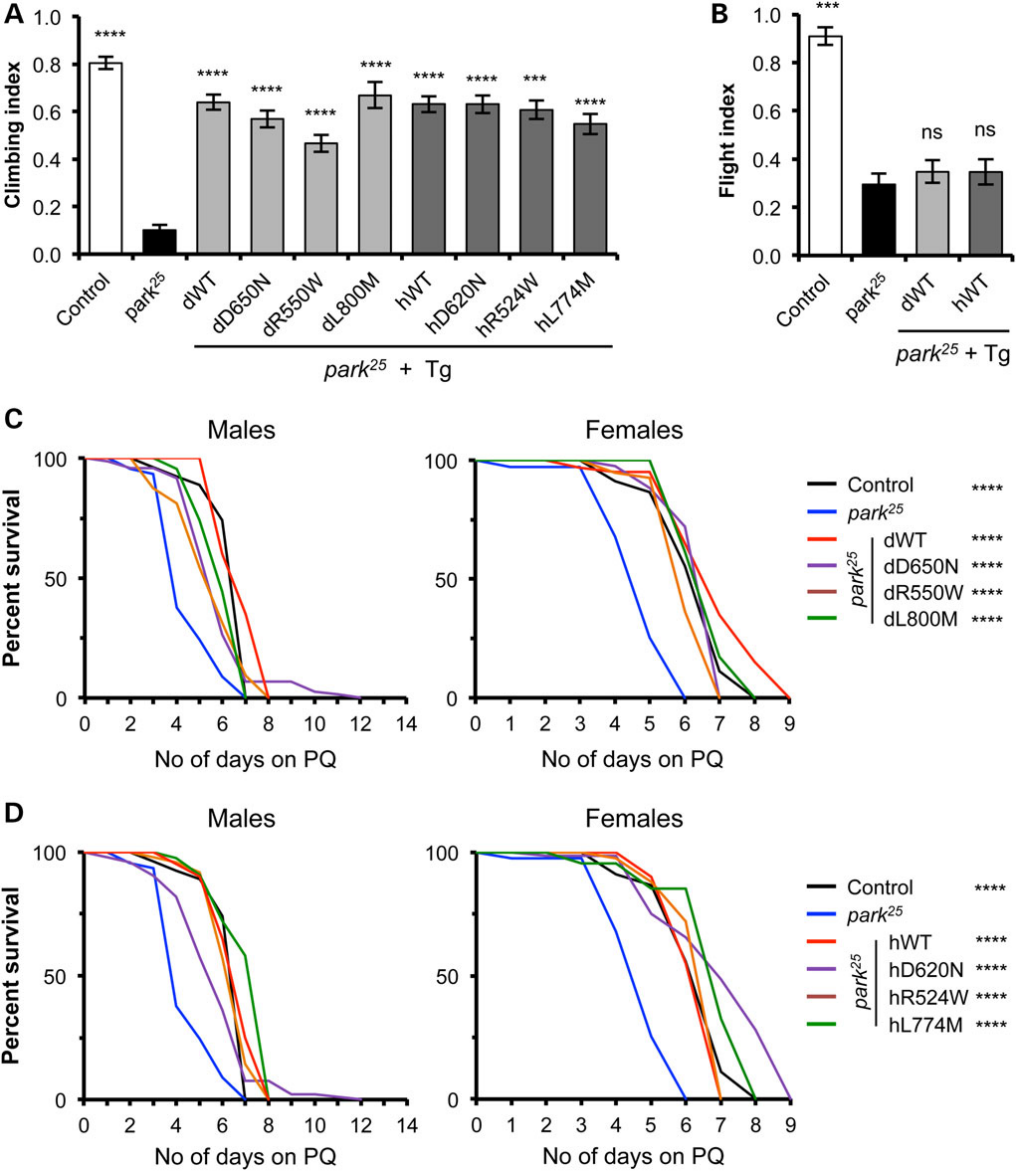
Overexpression of *vps35* rescues some *parkin*-mutant phenotypes. Ubiquitous overexpression of all variants of *Drosophila* or human *vps35* rescues *parkin*-mutant climbing defect (**A**), but not the flight defect (**B**). *n* = 50–150 animals. Sensitivity of *parkin* mutants to 5 mm paraquat (PQ) exposure was completely rescued by transgenic expression of all variants of *Drosophila* (**C**) or human (**D**) *vps35. n* = 40–100 animals. Control genotype is heterozygous *da-GAL4* driver line alone. Histograms show mean ± SEM. Statistical analysis: ns = not significant, **P < 0.01, ***P < 0.001 and ****P < 0.0001; one-way ANOVA with Bonferroni's correction, or Log-rank test for survival. All comparisons are with *park^25^*.

Since combining loss of function mutations caused the appearance of pathogenic phenotypes, we reasoned that overexpression of either *vps35* or *parkin* may ameliorate mutant phenotypes of the other gene. *vps35* mutants are developmental, lethal and present characteristic melanotic masses in larvae (7). Strong, ubiquitous overexpression of *parkin* in *vps35* mutants was unable to rescue these phenotypes (data not shown). However, we found that overexpression of *vps35* significantly rescued many of the *parkin* phenotypes, including the climbing defect and sensitivity to paraquat (Fig. 5A, C and D), with all variants of either *Drosophila* of human Vps35 providing an equivalent degree of rescue. In contrast, the flight defect in *parkin* mutants was not suppressed by *vps35* overexpression (Fig. 5B).

**Figure 6.**
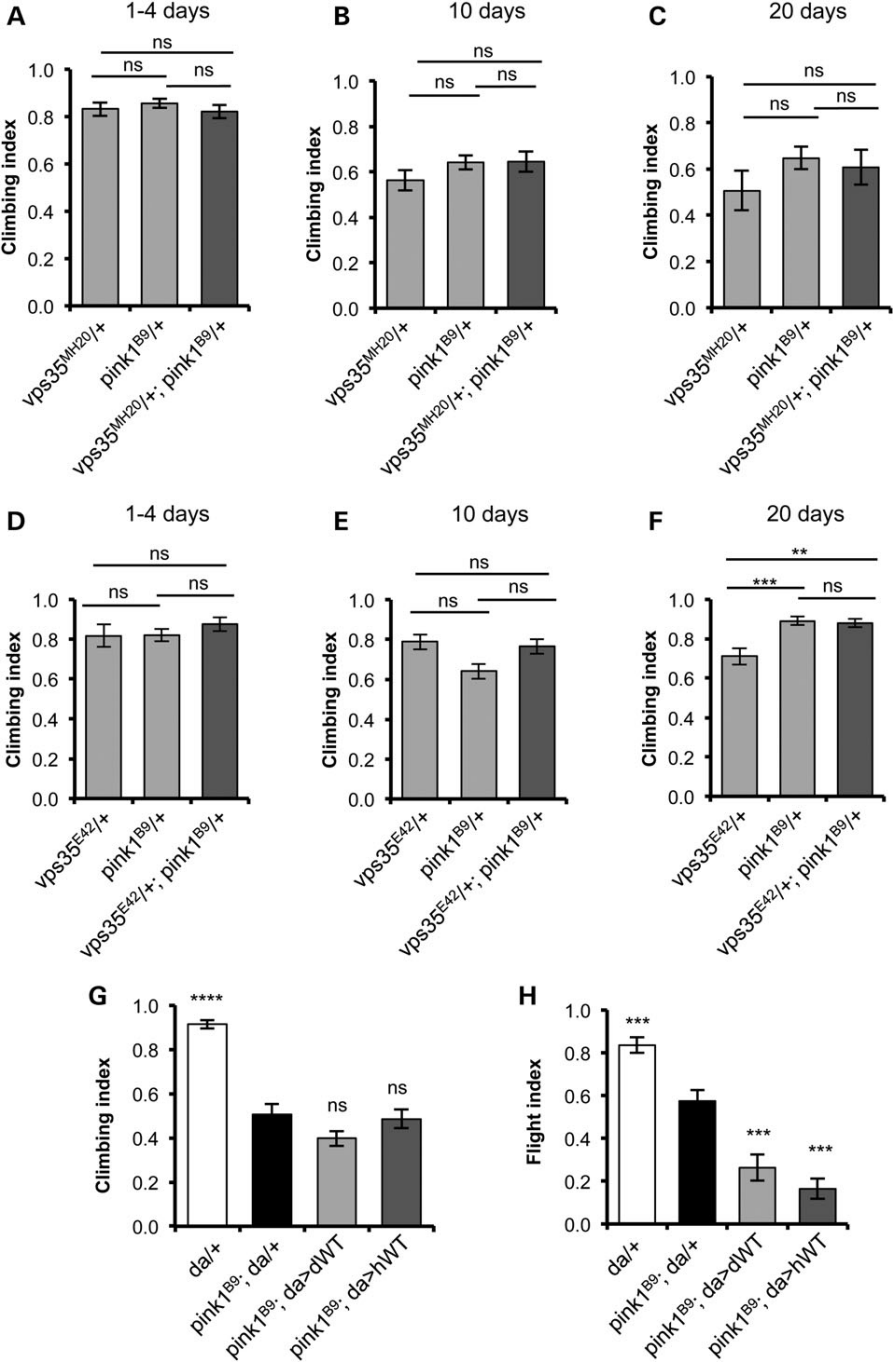
*vps35* does not genetically interact with *pink1*. (**A–F**) Climbing ability was assessed at different ages in animals single and double heterozygous for null alleles of *pink1* (*pink1^B9^*) and two independent null alleles of *vps35*, *vps35^MH20^* (A–C) and *vps35^E42^* (D–F). *n* = 77–94 (A), 53–87 (B), 27–42 (C), 23–39 (D), 50–85 (E) and 42–73 (F). (**G**) Overexpression of *vps35* does not suppress climbing deficits in *pink1* mutants (*n* = 60–100 animals) but slightly enhances the flight defect (**H**) (*n* = 35–60 animals). Histograms show mean ± SEM. Statistical analysis: ns = not significant, **P < 0.01, ***P < 0.001 and ****P < 0.0001; one-way ANOVA with Bonferroni's correction. Comparisons are with *pink1*-mutant control (*pink1^B9^*; *da(GAL4)*/+) unless otherwise indicated.

Substantial evidence supports a role for PINK1 and Parkin to act, at least partly, in a common pathway, so we also tested for genetic interaction between *vps35* and *pink1*. In contrast to the previous results, we found no synergy in *vps35/+;pink1/+* double heterozygotes, even after ageing (Fig. 6A–F). We also found *vps35* overexpression did not rescue *pink1* phenotypes (Fig. 6G, H and data not shown), nor did *pink1* overexpression alter *vps35* phenotypes (data not shown). In fact, while vps35 overexpression did not alter the *pink1* climbing defect, it actually worsened the flight defect (Fig. 6H). Thus, while the preceding results indicate a strong genetic interaction between *vps35* and *parkin*, surprisingly, there appears to be no genetic interaction between *vps35* and *pink1*.

## Discussion

Mutations in *VPS35* cause a late-onset, autosomal dominant form of PD. Replicating genetic mutations in classic model systems has been a powerful approach in illuminating the pathogenic mechanisms. One key issue in the molecular understanding of pathogenicity, especially with mutations causing dominant forms of disease, is to determine the basic mode of toxicity that can be broadly predicted to be toxic gain of function, dominant negative and/or haploinsufficiency through partial loss of function.

Current literature analysing the impact of the D620N mutation on the cell biological function of the retromer complex presents conflicting results as to whether endosome-to-TGN and/or endosome-to-plasma membrane recycling is affected (14–16). However, despite discrepancies on which trafficking routes may be perturbed, there is a general consensus that D620N has impaired function compared with WT without overt dominant toxicity (14–16,23). Evidence for the molecular basis of this dysfunction indicates that D620N specifically disrupts the recruitment of the WASH complex to endosomes by decreased binding of VPS35 to the WASH component FAM21 (15,16). These findings argue for partial loss of function from this pathogenic mutation, rather than toxic gain of function. Our results largely support these findings. First, our data provide clear evidence that transgenic expression of WT or variant forms of *vps35* does not cause overt toxicity. Second, all variants of Vps35 appear to function near normally as they are able to substantially rescue *vps35-*mutant phenotypes, including viability, NMJ morphology and Wg secretion. Interestingly, while the WT form of both *Drosophila* and human Vps35 could substantially rescue a larval locomotor defect, the variant forms could only provide partial rescue consistent with a partially reduced functionality. Furthermore, although the pathogenic dD650N variant could rescue *vps35* mutants to adult viability, these animals revealed a distinct locomotor deficit in contrast to the WT and non-pathogenic dL800M. The locomotor activity assays provide a highly sensitive read-out of the complex neuromuscular circuit. Even small disturbances in function at any part of this circuit will result in a quantifiable deficit. Thus, the failure of the Vps35 pathogenic variants to fully rescue this phenotype likely reflects a subtle decrease in functionality.

A recent study of lentiviral-mediated overexpression of VPS35 in cortical neurons reported neurotoxic effects *in vitro* and *in vivo*; however, the effects of D620N were often similar to that of overexpressing WT VPS35 (17). Thus, although it was proposed that where the effect of D620N overexpression differed from overexpression of WT, this was due to some gain-of-function toxicity, the effects could also result from the reduced function of D620N acting as a partial dominant negative when overexpressed.

The disruption of VPS35 D620N binding to FAM21 suggests a plausible pathogenic mechanism since this appears to affect the trafficking of the autophagy protein ATG9A and subsequent induction of autophagy (16). Since autophagy is a key mechanism for degradation of cellular components such as long-lived proteins, large aggregates and defective organelles, autophagy inhibition is considered a major culprit in the pathogenesis of neurodegenerative diseases including PD. It is notable that VPS35 haploinsufficiency has been reported to enhance neuropathology in a mouse model of Alzheimer's disease (24), where amyloid plaques and neurofibrillary tangles highlight decreased autophagy as a prominent mechanism for this disease (25). In this context, it is highly plausible that the partial loss of function conferred by *VPS35* mutations may lead to a haploinsufficiency of VPS35 function related to autophagy induction in PD as well.

Partial loss of function could also affect other VPS35 functions. To date, while there is very limited evidence linking ‘classic’ retromer-mediated endosomal retrieval in PD, mitochondrial dysfunction has long been considered a key pathogenic mechanism (26). Our genetic studies of *vps35* with *parkin* and *pink1* support an involvement in Vps35 regulating mitochondrial homeostasis but have yielded some surprising results. First, we uncovered a clear genetic interaction between *vps35* and *parkin* consistent with the two genes functioning in a common process. Parkin has been linked to the regulation of many cellular processes (27), but by far the most prominent is the autophagic degradation of damaged mitochondria, or mitophagy, a mechanism of mitochondrial quality control (28). Recently, it was shown that an early mechanism of Parkin-mediated mitochondrial homeostasis was in the generation of MDVs (22), previously also linked to VPS35 function (19). Clearly, more work is needed to elucidate the precise mechanisms linking *vps35* function to *parkin*; however, our results are consistent with Vps35 and Parkin cooperating in such a mitochondrial quality control process and provide the first *in vivo* evidence for a functional interaction.

Given this robust *vps35-parkin* genetic interaction, the lack of evidence for a genetic interaction between *vps35* and *pink1* was surprising given the substantial body of evidence linking the functions of PINK1 and Parkin (28), and the fact that PINK1 also appears to promote MDV formation (22). Nevertheless, this result adds further weight to the growing evidence that PINK1 and Parkin have independent as well as common functions. Most notably, PINK1 has been suggested to regulate the activity of Complex I of the respiratory chain independent of its role in mitophagy or Parkin (29,30).

The characterization of these new *Drosophila* models of PD provides *in vivo* evidence that dominant mutations in *VPS35* likely do not act via a dominant toxic gain of function but rather through a partial loss of function conferring an age-dependent haploinsufficiency. Moreover, our genetic studies provide further support for *vps35* deficiency impacting on mitochondrial quality control processes. Further work will be needed to understand the full spectrum of VPS35 and retromer function on cellular homeostasis, but these models provide a platform with which to elucidate this in the context of the ageing brain.

## Material and Methods

### *Drosophila* genetics

*Drosophila* were raised under standard conditions at 25°C on food consisting of agar, cornmeal and yeast unless otherwise stated. The UAS-*hVPS35* transgenes were generated by sub-cloning human *VPS35* variants, kindly provided by T. Strom, into pUASTattB vector using primers introducing 5′ NotI and 3′ XhoI cloning sites. The UAS-*dVps35* transgenes were similarly generated by sub-cloning the full length *Drosophila Vps35* cDNA (clone SD03023 from the *Drosophila* Genomics Resource Center) into pUASTattB. Variants of *dVps35* were generated by standard site-directed mutagenesis prior to sub-cloning. All constructs were verified by sequencing. Transgenic lines were made using the phiC31 method using target strain PBac{y[+]-attP-3B}VK00031, integrating the transgene at cytological location 62E1 (BestGene, Inc.). At least three lines of each variant were initially tested for consistency before focussing on a single line.

The *vps35^MH20^* and *vps35^E42^* alleles and UAS-*vps35-HA* have been described before (7,8) and were kindly provided by J.-P. Vincent (Crick Institute) and K. Basler (IMLS, Zurich). *pink1^B9^* mutants (31) were kindly provided by J. Chung (SNU). *park^25^* mutants have been described before (32). Stocks to analyse *vps35* function in wing discs, *hh*-*GAL4*, UAS-*red fluorescent protein* and *Ubx-FLP*; FRT42D *arm-lacZ* were kindly provided by D. Strutt. *w^1118^*, *da-GAL4* and *GMR-GAL4* strains were obtained from the Bloomington *Drosophila* Stock Center (Bloomington, IN).

### Behavioural assays

Fertility tests, flight and climbing assays were performed as previously described (32,33), with only males assessed here. Larval crawling was performed using animals grown at 29°C. Third instar larvae expressing the indicated *vps35* transgene using *da*-*GAL4* in the *vps35*-mutant background were collected from vials, briefly rinsed with distilled water and placed on 2% agar. Movies were captured, which were then analysed manually, recording the peristaltic waves per 2 min for each larva.

For lifespan analysis, animals were raised in low density, and shortly following eclosion, 40–100 adult flies were separated by gender and kept in groups of 15 flies per vial. The number of deaths was recorded every 2 or 3 days as the flies were transferred to new media. Survival upon exposure to paraquat was performed similarly, except flies were kept in vials with filter paper soaked with 5 mm paraquat in 5% sucrose. Survival was analysed by Log-rank (Mantel–Cox) test. For all the experiments, unless otherwise indicated, only males were used.

### Immunoblotting

Proteins were prepared in lysis buffer (50 mm Tris–HCl, 150 mm NaCl, 10% [vol/vol] glycerol, 1% Triton X-100, 10 mm *N*-ethylmaleimide, 2 mm ethylene glycol tetraacetic acid, 1 mm, MgCl_2_, 50 µm MG-132 and protease inhibitor mixture [Roche]), and 60 µg were loaded on SDS-PAGE gels. After resolving, proteins were transferred onto PVDF membrane. Membranes were blocked by 5% skimmed milk, 1% foetal bovine serum (FBS) in Tris-Buffered Saline with 0.1% Tween 20. Rabbit anti-VPS35 (Proteintech 10236–1-AP, 1:500) was incubated in blocking solution overnight at 4°C. Mouse anti-Actin (Millipore MAB1501, 1:10 000) was incubated in blocking solution at room temperature (RT) for 1 h. Membranes were washed repeatedly in phosphate-buffered saline (PBS)–0.1% Tween 20 (PBST), and then appropriate horseradish peroxidase (HRP)-conjugated secondary antibodies were incubated for 1 h at RT. Detection was achieved with ECL-Plus detection kit (Amersham) using radiographic film.

### Immunohistochemistry

Wandering third instar larvae were selected for NMJ analysis essentially as previously described (34). Larval body wall muscles were dissected in a saline containing 128 mm NaCl, 2 mm KCl, 4 mm MgCl_2_, 0.1 mm CaCl_2_, 35.5 mm sucrose and 5 mm Hepes (pH 7.2), and fixed in a freshly prepared ice-cold 4% paraformaldehyde for 20 min.

The preparations were then washed in PBST for three times in 15 min, followed by 1 h blocking in 5% FBS in PBST. Samples were incubated overnight at 4°C with anti-HRP antibody (Jackson Immunoresearch Laboratories, used at 1:200) and anti-discs large (4F3, Developmental Studies Hybridoma Bank, used at 1:300). Secondary antibodies (Invitrogen) AlexaFluor 594 goat anti-rabbit (1:500) and AlexaFluor 488 goat anti-mouse (1:500) were used, respectively.

Quantification of bouton number was performed on longitudinal muscle 6/7 at segment A2 as previously described (35). Total number of boutons (Type 1B and 1S) was counted on Olympus FV1000 microscope under Plan Apo 60X (N.A. 1.42) oil immersion lens. The number of boutons was normalized to the respective muscle surface area. Muscles were photographed at a 10× magnification, and the area was quantified using Image J software.

Wing discs were analysed essentially as previously described (36). Wandering third instar larvae were dissected and fixed for 1 h at RT in 4% paraformaldehyde. They were then washed twice for 15 min with PBS containing 0.1% Triton X-100 (PBST), blocked for 1 h with PBS with 1% bovine serum albumin. The samples were then incubated with anti-Wg (1:40; Developmental Studies Hybridoma Bank, Iowa) and anti-β-gal (1:1000, Cappel) in blocking buffer overnight at 4°C with rotation. The next day, the samples were washed twice with PBST for 15 min each, following which they were incubated with AlexaFluor-488 goat anti-mouse antibody and AlexaFlour-546 anti-rabbit antibody for 1 h. The samples were then washed twice for 15 min each with PBST, following which the wing discs were mounted in Mowiol and stored at 4°C until imaging. Images were captured using Nikon Eclipse T1 confocal microscope with a 40× (N.A. 1.3) oil immersion objective.

For quantification of Wg levels, following projections of Z-stacks for each wing disc, regions of interest (ROIs) were marked inside the clone and ROIs of the same dimensions were marked on the neighbouring WT tissue. Measurement of intensities of Wg staining was obtained, and the intensity per unit area was calculated for each of the mutant and WT ROI. Data were analysed using paired Student's *t*-test.

Dopaminergic neurons were analysed as described previously (37). Anti-tyrosine hydroxylase (Chemicon AB152) was used at 1:200. Quantification was done with the experimenter blind to the genotype of the samples.

### Statistical analysis

Calculations and statistical analysis were performed using Excel (Microsoft Corporation, Redmond, WA) and Prism software (GraphPad Software, Inc., San Diego, CA). Statistical significance was determined by one-way ANOVA with Bonferroni's correction for multiple comparisons or by Student's *t*-test. All data shown were obtained from at least three independent experiments. Lifespan and paraquat survival were analysed by Log-rank (Mantel–Cox) test.

## Supplementary Material

Supplementary Material is available at *HMG* online.

## Funding

The work was funded by a Wellcome Trust/Medical Research Council (MRC) Parkinson's Disease Consortium grant to UCL/IoN, the University of Sheffield and the MRC Protein Phosphorylation Unit at the University of Dundee (grant number WT089698), an European Research Council (ERC) Starting Grant (no. 309742) and a project grant (G-1201) from Parkinson's UK. The Wellcome Trust is acknowledged for support of the Sheffield Light Microscopy Facility (GR077544AIA). The funders had no role in study design, data collection and analysis, decision to publish, or preparation of the manuscript. Funding to pay the Open Access publication charges for this article was provided by the Wellcome Trust, ERC and Parkinson's UK.

## Supplementary Material

Supplementary Data
