# Supplementary material for: VPS35 pathogenic mutations confer no dominant toxicity but partial loss of function in Drosophila and genetically interact with parkin
Source: Hum Mol Genet. 2015 Aug 6;24(21):6106–17. doi: 10.1093/hmg/ddv322 (PMC4599670; doi:10.1093/hmg/ddv322)
Supplement: Supplementary Data [file supp_24_21_6106__index.html]

VPS35 pathogenic mutations confer no dominant toxicity but partial loss of function in Drosophila and genetically interact with parkin — VPS35 pathogenic mutations confer no dominant toxicity but partial loss of function in Drosophila and genetically interact with parkin — VPS35 pathogenic mutations confer no dominant toxicity but partial loss of function in Drosophila and genetically interact with parkin — Supplementary Data 

# *VPS35* pathogenic mutations confer no dominant toxicity but partial loss of function in *Drosophila* and genetically interact with *parkin*

## Supplementary Data

Supplementary Data

- Supplementary Data - Pdf file
