# Supplementary material for: VPS35 pathogenic mutations confer no dominant toxicity but partial loss of function in Drosophila and genetically interact with parkin
Source: Hum Mol Genet. 2015 Aug 6;24(21):6106–17. doi: 10.1093/hmg/ddv322 (PMC4599670; doi:10.1093/hmg/ddv322)
Supplement: Supplementary Data [file supp_ddv322_ddv322supp.pdf]

Supplementary Figure 1

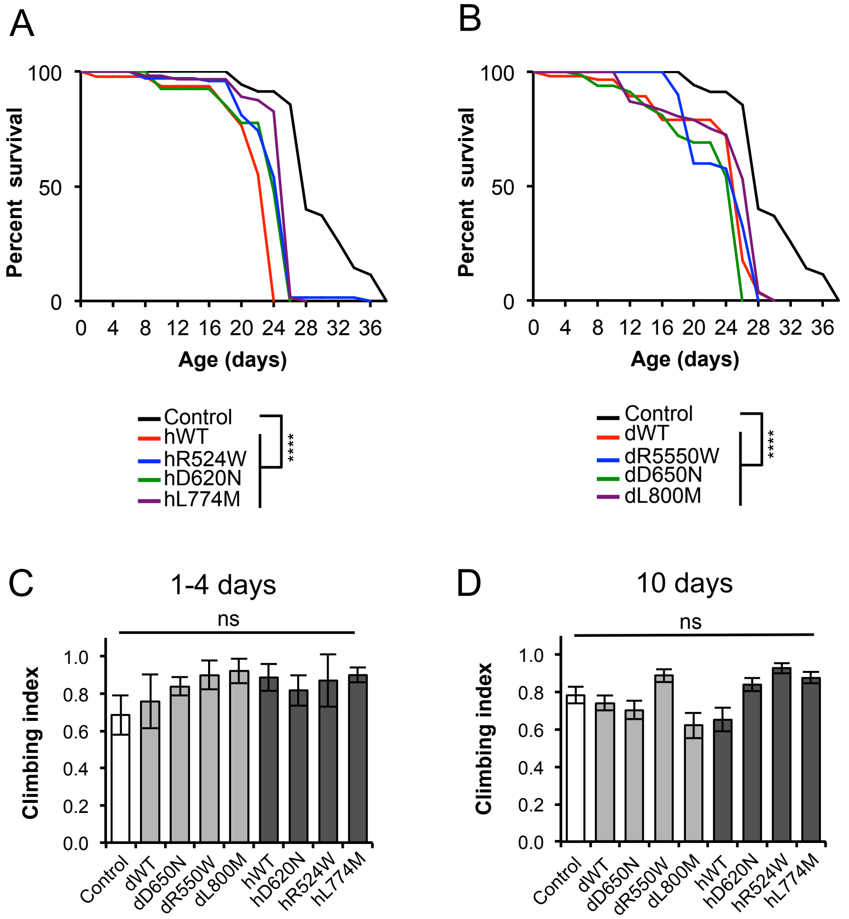

No dominant toxicity of human or *Drosophila* Vps35 variants raised at 29°C. (A, B) Ubiquitous transgene expression via *da-GAL4* modestly shortens lifespan but is affected similarly by all Vps35 variants (**** P < 0.0001, n = 40-100 animals). (C, D) Transgene expression does not affect climbing ability with age (all not significant (ns) compared to control). n = 15-30 (C), 35-55 (D). Histograms show mean ± s.e.m. Statistical analysis was Log-rank test for lifespan and one-way ANOVA with Bonferroni correction for climbing. Control genotypes are heterozygous *GAL4* driver line alone.
